# Korat‐Scrub Typhus Score: A Clinical Tool for Predicting Scrub Typhus in Patients With Acute Undifferentiated Febrile Illness

**DOI:** 10.1111/tmi.70154

**Published:** 2026-05-01

**Authors:** Wilawan Thipmontree, Ekkarat Wongsawat, Saowaluk Silpasakorn, Yupin Suputtamongkol

**Affiliations:** ^1^ Department of Medicine Maharat Nakhon Ratchasima Hospital Nakhon Ratchasima Thailand; ^2^ Faculty of Medicine Siriraj Hospital Mahidol University Bangkok Thailand

**Keywords:** AUFI, predictor score, scrub typhus

## Abstract

**Objective:**

Scrub typhus is a major cause of acute undifferentiated febrile illness (AUFI) in the Asia–Pacific region. Nonspecific presentation, limited diagnostic accuracy and delayed treatment increase morbidity and mortality. This study aimed to develop a simple clinical score for early diagnosis in adults with AUFI.

**Methods:**

A cross‐sectional study of adults (≥ 18 years) with AUFI was conducted at a tertiary hospital in northeastern Thailand from June 2021 to May 2022. The Korat–Scrub Typhus Score was developed by analysing all clinically relevant variables using multivariable logistic regression with backward stepwise selection. Model performance was assessed using the Hosmer–Lemeshow goodness‐of‐fit test and the area under the receiver operating characteristic curve (AUC), with internal validation via 1000 bootstrap replications.

**Results:**

Among 366 AUFI patients, 75 had scrub typhus. Most were male (66.7%), with mean age of 53.6 ± 17.6 years and median illness duration of 4 days (IQR 2–7). Five predictors were identified: outdoor activities, rash (excluding eschar), headache, abnormal chest radiograph findings and aspartate aminotransferase ≥ 100 U/L. The Korat‐Scrub Typhus Score (0–13) stratified risk into low (< 5), moderate (5–6) and high (> 6) groups, with likelihood ratios of 0.55 (95% CI, 0.43–0.71), 2.20 (95% CI, 1.37–3.53) and 9.22 (95% CI, 4.19–20.25), respectively. The model showed good calibration, acceptable discrimination (AUC = 0.783, 95% CI 0.725–0.842) and minimal overfitting after internal validation.

**Conclusion:**

Scrub typhus is a common cause of AUFI. The Korat‐Scrub Typhus Score uses simple clinical and laboratory data to support early diagnosis and timely treatment.

## 1 Introduction

Scrub typhus, a vector‐borne disease transmitted by chigger mites, is a common cause of acute undifferentiated febrile illness (AUFI) [1]. Its incidence increased in Asia from 2000 to 2018 [2] but declined during the COVID‐19 era [3]. Clinical manifestations overlap with dengue, leptospirosis, malaria and primary bacteremia [4]. Although the presence of an eschar is pathognomonic, its prevalence varies widely across regions (1%–97%) [5].

Disease severity ranges from mild febrile illness—characterised by fever, headache and myalgia—to severe multiorgan failure, including pneumonitis, acute respiratory distress syndrome, renal failure, meningoencephalitis, septic shock, disseminated intravascular coagulation, myocarditis and pericarditis [1, 5]. Doxycycline and azithromycin are the mainstay treatments [6, 7]. Mortality was 6.0% (0%–70.0%) without treatment and 1.4% (0%–33.3%) with treatment [8, 9]. The diagnostic gold standard for scrub typhus is serologic testing, including the indirect immunofluorescence assay (IFA) and the indirect immunoperoxidase (IIP) test. Both methods have low sensitivity in acute‐phase sera and require paired samples to confirm. The IFA requires specialised equipment and technical expertise, whereas the IIP can be performed with standard laboratory tools but still demands technical skill. Molecular assays are accurate but technically complex and costly, whereas rapid serologic tests offer simpler alternatives with variable performance. No reliable rapid commercial antigen test is currently available for scrub typhus. The Rathi–Goodman Score (RGA) was initially developed in India to predict spotted fever in patients under 20 years using 20 parameters [10] and later validated for diagnosing scrub typhus in children aged 6 months to 12 years [11]. This study aimed to develop a simple and practical risk score to facilitate early diagnosis of scrub typhus in adults with AUFI.

## 2 Methods

### 2.1 Study Design and Study Populations

A cross‐sectional study was conducted among adults (≥ 18 years) presenting with acute undifferentiated febrile illness (AUFI) at Maharat Nakhon Ratchasima Hospital, a 1500‐bed tertiary care centre in Nakhon Ratchasima Province (Korat), northeastern Thailand. AUFI was defined as a fever of ≥ 38°C lasting less than 14 days without organ‐specific symptoms. A suspected scrub typhus case was defined as an AUFI patient who underwent a point‐of‐care IgM test for scrub typhus using an immunochromatographic test (STANDARD Q, SD BIOSENSOR). Patients with suspected scrub typhus between June 1, 2021 and May 31, 2022, were included in the analysis. Patients with confirmed dengue or malaria were excluded. All patients underwent the following investigations:

- Indirect immunofluorescence assay (IFA) for detection of IgM and IgG antibodies against pooled antigens of *Orientia tsutsugamushi* strains Karp, Kato and Gilliam, as well as *Rickettsial typhi* and *Leptospira* spp., using paired sera collected 7–14 days apart.
- Polymerase chain reaction (PCR):
  ○ Detection of the 47‐kDa and 56‐kDa genes of O. tsutsugamushi [12, 13].
  ○ Detection of R. typhi DNA.
  ○ Detection of the LipL32 gene of Leptospira.
  ○ Detection of Flaviviruses RNA
- Dengue nonstructural protein 1 (NS1) antigen (Bioline, Abbott Diagnostics Korea Inc.).
- Blood cultures for bacterial pathogens.
- Urine culture is indicated when the urine analysis shows WBC > 10 cells/HPF.
- Baseline laboratory test: complete blood count, blood urea nitrogen, serum creatinine, electrolyte, liver function test, urine analysis, chest radiography.

### 2.2 Definition

Scrub typhus was confirmed if patients met any of the following criteria: (1) positive PCR targeting the 47‐kDa and/or nested PCR targeting the 56‐kDa genes of *O. tsutsugamushi* ; (2) a fourfold rise in IgM or IgG antibody titre using IFA [12, 14]; or (3) a single IFA with IgM antibody titre ≥ 1:400 [15].

Unknown AUFI was defined as cases in which all of the above tests were negative.

### 2.3 Sample Size

The study aims to develop a predictive model for scrub typhus using key clinical and physical examination variables, expecting an AUC of not less than 75%. The prevalence of scrub typhus is approximately 20%. Based on this, the study plans to use around five predictors. Sample size estimation using STATA indicated that at least 330 participants are required, yielding an Events per Predictor Parameter (EPP) of 13.2 and a shrinkage factor of 0.9, which should result in a well‐performing model.

### 2.4 Statistical Analysis, Risk Factors Score Development and Internal Validate

Descriptive statistics, including means and percentages, were used to summarise baseline characteristics. Between‐group comparisons were performed using the Fisher exact test for categorical variables and the Student *t*‐test for normally distributed continuous variables. A two‐tailed *p* value < 0.05 was considered statistically significant.

All clinically relevant variables were initially included in the multivariable logistic regression model. A backward stepwise selection procedure was then applied to refine the model by excluding noncontributory predictors. Among the candidate models generated, the final model was chosen based on both statistical performance (e.g., discrimination, calibration and model fit criteria) and clinical plausibility. The final predictive score was derived by rescaling the beta coefficients and rounding them to the nearest integer or 0.5 to maintain close approximation to the original values while ensuring simplicity for clinical use. The accuracy of this predictive score was assessed in terms of calibration and discrimination. Calibration was evaluated using the Hosmer–Lemeshow goodness‐of‐fit test and a calibration plot was presented to compare the agreement between the probability of scrub typhus estimated by the predictive score and the observed cases. Discriminative ability was assessed by the area under the receiver operating characteristic curve (AUC). Internal validation was conducted using a bootstrapping resampling procedure with 1000 replicates. For clinical applicability, the total score was classified into three risk categories (low, moderate and high risk). The predictive performance of each category was summarised using positive predictive value (PPV) and likelihood ratio (LR).

## 3 Results

**FIGURE 1:**
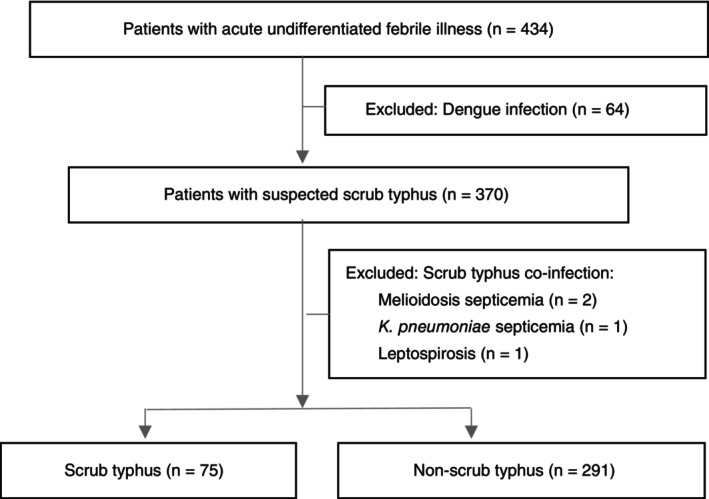
Flow of patients with acute undifferentiated febrile illness.

Of 370 adults (≥ 18 years) with acute undifferentiated febrile illness (AUFI) and suspected scrub typhus, 79 were confirmed. After exclusion of four co‐infected cases ( *Burkholderia pseudomallei* septicemia, *n* = 2; *Klebsiella pneumoniae* septicemia, *n* = 1; and leptospirosis, *n* = 1), 75 patients were included in the final analysis. Diagnosis was confirmed by polymerase chain reaction (PCR) (*n* = 29), PCR with a fourfold rise in indirect immunofluorescence assay (IFA) titre (*n* = 19), a fourfold IFA rise alone (*n* = 12), or a single IgM titre ≥ 1:400 (*n* = 15) (Figure 1). Eschar was observed in 15 patients (20%), most commonly on the abdomen and upper chest. The case fatality rate was 10%, mainly attributable to multiorgan failure with acute respiratory distress syndrome, compounded by delayed diagnosis and treatment.

Overall, 366 patients were included in the analysis: 75 with scrub typhus and 291 with other causes of AUFI (Table 1). The nonscrub typhus group comprised melioidosis septicemia (*n* = 21), primary bacteremia (*n* = 18), leptospirosis (*n* = 15), murine typhus (*n* = 7), chikungunya (*n* = 4), acute retroviral syndrome (*n* = 2), acute hepatitis B (*n* = 1), influenza A infection (*n* = 1) and cases of unknown AUFI (*n* = 212). Most patients were male (66.7%), with a mean age of 53.6 ± 17.6 years and a median illness duration of 4 days (interquartile range, 2–7). The rapid immunochromatographic test (ICT) demonstrated a sensitivity of 74.7%, a specificity of 71.8%, a positive predictive value of 40.6% (95% CI, 35.3–46.1) and a negative predictive value of 91.7% (95% CI, 88.1–94.2). False‐positive results occurred in patients with leptospirosis, melioidosis, chikungunya, *Streptococcus suis* septicemia, *K. pneumoniae* septicemia and influenza A infection.

**TABLE 1:** Baseline characteristics of 366 patients with acute undifferentiated febrile illness.

Factors	Total (*n* = 366, %)	Scrub typhus (*n* = 75, %)	Non‐scrub typhus (*n* = 291, %)	*p*
Male	244 (66.7)	51 (68.0)	193 (66.3)	0.891
Age ≥ 60 years	151 (41.3)	37 (49.3)	114 (39.2)	0.116
Duration of illness ≤ 7 days	313 (85.5)	63 (84.0)	250 (85.9)	0.713
Outdoor activities^a^	128 (35.0)	45 (60.0)	83 (28.5)	< 0.001
Comorbidities				
Alcoholism	88 (24.0)	19 (25.3)	69 (23.7)	0.763
Diabetes mellitus	84 (22.9)	17 (22.7)	67 (23.0)	1.000
Liver cirrhosis	19 (5.2)	2 (2.6)	17 (5.8)	0.386
Clinical manifestations				
Headache	94 (25.7)	26 (34.7)	68 (23.4)	0.054
Myalgia	159 (43.4)	35 (46.7)	124 (42.6)	0.602
Eschar	15 (4.1)	15 (20.0)	0	< 0.001
Rash (excluding eschar)	20 (5.5)	10 (13.3)	10 (3.4)	0.014
Conjunctivitis	19 (5.2)	8 (10.7)	11 (3.8)	0.035
Lymphadenopathy	2 (0.5)	1 (1.33)	1 (1.33)	0.368
Laboratory findings				
WBC ≥ 10 × 10^9^/L	207 (56.6)	45 (60.0)	162 (55.7)	0.517
Platelet < 100 × 10^9^/L	112 (30.6)	26 (34.7)	86 (29.5)	0.402
AST ≥ 100 U/L	149 (40.7)	46 (61.3)	103 (35.4)	< 0.001
ALT ≥ 100 U/L	86 (23.5)	28 (37.3)	58 (19.9)	0.002
ALP ≥ 200 U/L	90 (24.6)	28 (37.3)	62 (21.3)	0.006
Urine protein ≥ 1+	191 (52.2)	40 (53.3)	151 (51.9)	0.897
Urine RBC ≥ 10 per HPF	129 (35.2)	30 (40.0)	99 (34.0)	0.345
Urine WBC ≥ 10 per HPF	60 (16.4)	14 (18.7)	46 (15.8)	0.600
Abnormal chest radiograph	40 (10.9)	19 (25.3)	21 (7.2)	< 0.001
Interstitial infiltration		19 (100)	10 (47.6)	
Alveolar infiltration		0	11 (52.4)	
Outcome: Death	41 (11.2)	10 (13.3)	31 (10.6)	0.539

Abbreviations: ALP, alkaline phosphatase; ALT, alanine aminotransferase; AST, aspartate aminotransferase; HPF, high‐power field; RBC, red blood cell; U/L, units per litre; WBC, white blood cell.

^a^ Outdoor activities: activities with potential chigger exposure (e.g., farming, trekking, contact with vegetation).

All clinically relevant variables were entered into a multivariable logistic regression regardless of statistical significance. Backward stepwise selection was used to identify the final model, chosen for both statistical performance and clinical plausibility. The predictive score was derived by rescaling beta coefficients and rounding to the nearest integer or 0.5 for ease of use. Five predictors remained in the final model (*p* < 0.1): outdoor activities, rash (excluding eschar), headache, abnormal chest radiograph findings and aspartate aminotransferase (AST) ≥ 100 U/L (Table 2). The score was derived by dividing each significant coefficient by the smallest coefficient, resulting in a total range of 0–13. Higher scores were associated with an increased risk of scrub typhus (Table 2). Based on the score distribution, patients were classified into low (< 5), moderate (5–6) and high‐risk (> 6) groups, with corresponding likelihood ratios of 0.55 (95% CI, 0.43–0.71), 2.20 (95% CI, 1.37–3.53) and 9.22 (95% CI, 4.19–20.25) and positive predictive values of 12.5% (95% CI, 8.8–16.9), 36.2% (95% CI, 24.0–49.9) and 70.4% (95% CI, 49.8–86.2), respectively (Table 3).

**TABLE 2:** Development of the Korat–Scrub score for predicting scrub typhus in suspected cases.

Factors	OR (95% CI)	*p*	Beta coefficients	Scores
Outdoor activities^a^	3.56 (2.00, 6.34)	< 0.001	1.27	2.5
Headache	1.73 (0.93, 3.23)	0.086	0.55	1
Rash (excluding eschar)	7.90 (2.73, 22.88)	< 0.001	2.07	4
AST ≥ 100 U/L	3.45 (1.89, 6.27)	< 0.001	1.24	2.5
Abnormal CXR	4.71 (2.21, 10.01)	< 0.001	1.55	3

Abbreviations: AST, aspartate aminotransferase; CI, confidence interval; CXR, chest radiograph; OR, odds ratio.

^a^ Outdoor activities: activities with potential chigger exposure (e.g., farming, trekking and contact with vegetation).

**TABLE 3:** Clinical score to differentiate scrub typhus among patients with acute undifferentiated fever.

Risk categories	Score	Scrub typhus *n* = 75 (%)	Non‐scrub typhus, *n* = 291 (%)	PPV (95% CI)	LR+ (95% CI)	p
Low (*n* = 281)	< 5	35 (12.5)	246 (87.5)	12.5 (8.8, 16.9)	0.55 (0.43, 0.71)	< 0.001
Moderate (*n* = 58)	5–6	21 (36.2)	37 (63.8)	36.2 (24.0, 49.9)	2.20 (1.37, 3.53)	0.001
High (*n* = 27)	> 6	19 (70.4)	8 (29.6)	70.4 (49.8, 86.2)	9.22 (4.19, 20.25)	< 0.001

Abbreviations: LR+, positive likelihood ratio; PPV, positive predictive value.

### 3.1 Model Performance and Validation

**FIGURE 2:**
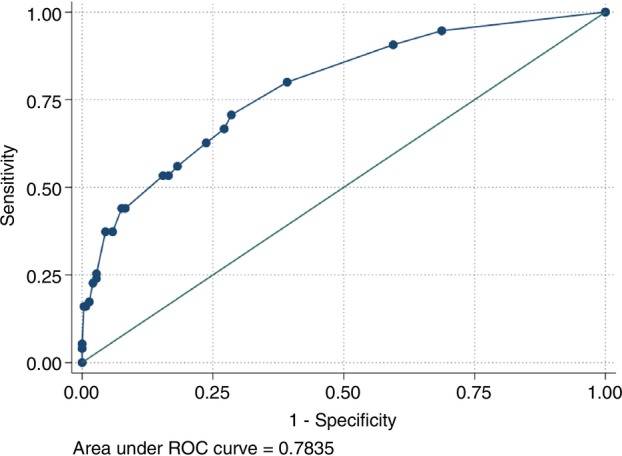
Receiver operating characteristic curve for predicting scrub typhus.

**FIGURE 3:**
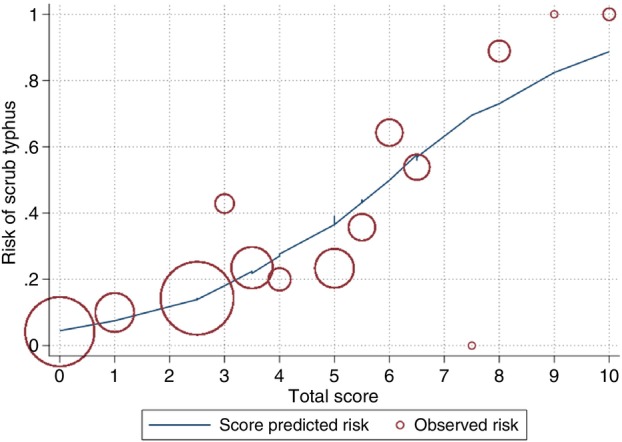
Calibration plot showing agreement between predicted and observed probability of scrub typhus.

Model calibration was good (Hosmer–Lemeshow χ^2^ = 3.61, *p* = 0.722) with close agreement between predicted and observed outcomes. Discrimination was acceptable (AUC = 0.783, 95% CI 0.725–0.842) (Figures 2, 3). Internal validation using 1000 bootstraps showed stable performance (optimism‐corrected C‐statistic = 0.771, 95% CI 0.717–0.831; calibration slope = 0.935; Brier scaled score = 18.1%), with minimal overfitting (shrinkage factor = 0.935).

## 4 Discussion

Scrub typhus is a major cause of acute undifferentiated febrile illness (AUFI) in the Asia–Pacific region, including Thailand [5]. The prevalence of scrub typhus in the present study was 21.4% (79/370), which was lower than in the previous decade (29.5%) [16] and consistent with other reports during the COVID‐19 era [3]. The disease typically presents with nonspecific symptoms that mimic other tropical infections, and delayed treatment can lead to severe complications and high mortality. Accurate and timely diagnosis is therefore crucial. However, diagnostic challenges remain, as serologic gold‐standard tests are imperfect. Polymerase chain reaction (PCR) is highly accurate in early disease but, in Thailand, is mainly available in university hospitals and research centres. Point‐of‐care serologic assays have limited sensitivity during the early stages of disease, with a meta‐analysis reporting 66% (95% CI: 0.37–0.86), due to the delayed development of antibody responses [17]. In this study, the POCT had 74.7% sensitivity, with false positives in patients with other AUFI causes. To facilitate early diagnosis, we developed the Korat–Scrub Typhus Score, a simple clinical tool to identify scrub typhus in adult patients (aged ≥ 18 years) presenting with AUFI. Dengue and malaria were excluded because reliable rapid diagnostic tests are routinely available in Thai hospitals. The score includes five predictors—rash (excluding eschar), abnormal chest radiograph findings, outdoor activities, AST ≥ 100 U/L and headache—with respective weights of 4, 3, 2.5, 2.5 and 1. A cutoff score > 6 yielded a likelihood ratio of 9.22 (95% CI: 4.19–20.25). Outdoor activities, particularly farming, were significant risk factors for chigger exposure. An eschar, a pathognomonic lesion resulting from a chigger bite, was detected in 20% of patients—consistent with previous Thai reports (8%–40.4%) [16]. Rash, predominantly maculopapular, was more frequent in the scrub typhus group (13.3%) than in the non–scrub typhus group (3.4%) (*p* = 0.014), although less common than the rate reported by the CDC in 2024 (25%–50%) [18].

Scrub typhus causes multisystem involvement through the vasculitis process affecting the brain, lungs, liver, kidneys and cardiovascular system [19]. Headache was a common but nonspecific symptom, and aseptic meningitis occurred in 10.7% of cases. Abnormal chest radiograph findings, reported in 42%–72% of Asian patients [20, 21, 22], were observed in 25.3% of our cases—lower than previously reported in our centre (43.6%) [16]. Bilateral interstitial infiltration was the most common finding, and a nonproductive cough was frequently observed. These findings suggest that scrub typhus should be considered in the differential diagnosis of atypical pneumonia. A systematic review reported hepatic involvement in 61.5% of cases, typically with AST and ALT levels exceeding twice the upper limit of normal [23]. In this study, AST and ALT > 100 IU/mL were significantly higher in the scrub typhus group than in the non–scrub typhus group; however, only AST was retained in the final model and included in the predictive score.

Mali et al. validated the Rathi–Goodman Score (RGA) as a diagnostic tool for scrub typhus in Indian paediatric patients. The RGA comprises four exposure risk factors, nine clinical (five rash‐related) and seven laboratory parameters, yielding an AUC of 0.850 (95% CI, 0.770–0.930) using IgM ELISA as the reference standard. However, C‐reactive protein, one of its parameters, is not routinely measured in AUFI [11]. The Korat‐Scrub Typhus Score, developed for adults, showed an AUC of 0.783 (95% CI, 0.725–0.842) using PCR and IFA confirmation. With only one exposure risk, two clinical and two basic laboratory parameters, it is simpler and more applicable in routine practice. Model performance and validation were satisfactory, showing good agreement between predicted and observed outcomes. The availability and performance of POCTs vary among countries and may depend on regional disease prevalence. In our study, combining the Korat‐Scrub Typhus Score with a POCT did not improve diagnostic accuracy (sensitivity 70.7%, specificity 71.5%).

This study has several limitations. First, it was conducted at a single centre. Second, the prevalence of scrub typhus was low. Third, the IFA used only combined antigens of *O. tsutsugamushi* Karp, Kato and Gilliam strains and may not have detected other strains, such as TA713, which are present in Thailand. Finally, the small number of convalescent samples limited interpretation of the IFA results.

In conclusion, Scrub typhus is a common aetiology of AUFI. The Korat‐Scrub Typhus Score facilitates early diagnosis and timely initiation of empirical antibiotics, helping to prevent disease progression and poor outcomes.

## Funding

Wilawan Thipmontree received funding from Maharat Nakhon Ratchasima Hospital.

## Ethics Statement

This study was approved by the Maharat Nakhon Ratchasima Hospital Institutional Review Board (MNRH IRB No. 029/2025). All procedures involving human participants were conducted in accordance with the principles of the Declaration of Helsinki. [Correction added on 22 May 2026, after first online publication: In the preceding section, the MNRH IRB number 187/2025 has been replaced with 029/2025.]

## Consent

The authors have nothing to report.

## Conflicts of Interest

The authors declare no conflicts of interest.

## Data Availability

The authors have nothing to report.
